# Use of complementary and alternative medicine by those with a chronic disease and the general population - results of a national population based survey

**DOI:** 10.1186/1472-6882-10-58

**Published:** 2010-10-18

**Authors:** Amy Metcalfe, Jeanne Williams, Jane McChesney, Scott B Patten, Nathalie Jetté

**Affiliations:** 1 University of Calgary, Department of Community Health Sciences TRW Building 3rd Floor 3280 Hospital Drive NW Calgary, Alberta, T2N 4Z6, Canada; 2 University of Calgary, Department of Clinical Neurosciences 1403 29 Street NW Calgary, Alberta, T2N 2T9, Canada; 3 University of Calgary, Department of Psychiatry 1403 29 Street NW Calgary, Alberta, T2N 2T9, Canada

## Abstract

**Background:**

The use of complementary and alternative medicine (CAM) is becoming more common, but population-based descriptions of its patterns of use are lacking. This study aimed to determine the prevalence of CAM use in the general population and for those with asthma, diabetes, epilepsy and migraine.

**Methods:**

Data from cycles 1.1, 2.1 and 3.1 of the Canadian Community Health Survey (CCHS) were used for the study. The CCHS is a national cross-sectional survey administered to 400,055 Canadians aged ≥12 between 2001-2005. Self-reported information about professionally diagnosed health conditions was elicited. CCHS surveys use a multistage stratified cluster design to randomly select a representative sample of Canadian household residents. Descriptive data on the utilization of CAM services was calculated and logistic regression was used to determine what sociodemographic factors predict CAM use.

**Results:**

Weighted estimates show that 12.4% (95% Confidence Interval (CI): 12.2-12.5) of Canadians visited a CAM practitioner in the year they were surveyed; this rate was significantly higher for those with asthma 15.1% (95% CI: 14.5-15.7) and migraine 19.0% (95% CI: 18.4-19.6), and significantly lower for those with diabetes 8.0% (95% CI: 7.4-8.6) while the rate in those with epilepsy (10.3%, 95% CI: 8.4-12.2) was not significantly different from the general population.

**Conclusion:**

A large proportion of Canadians use CAM services. Physicians should be aware that their patients may be accessing other services and should be prepared to ask and answer questions about the risks and benefits of CAM services in conjunction with standard medical care.

## Background

A variety of definitions of complementary and alternative medicine (CAM) have been reported in the literature. They range from "medical interventions that are not taught widely in medical schools or generally available in hospitals" [1] to "strategies that have not met the standards of clinical effectiveness, either through randomized controlled clinical trials or though the consensus of the biomedical community" [2]. Others avoid definitions and instead use a classification system that groups CAM practices into manual therapies, mind-body therapies, movement-based therapies, oral therapies, and support therapies [3].

Rates of CAM use vary widely in the literature from 6% to 84% [1-8], and broadly show that women [4,6,8-10], those with high incomes [3,4,9], those with high education levels [3,4,8-10], certain ethnic groups [6,7] and those with a chronic condition [4,7,8] are more likely to use CAM than the general population. CAM services are also widely reported to be used in conjunction with standard medical care [2,3,10,11], but the use of these services is not often disclosed to health professionals [12]. This lack of reporting is particularly concerning for patients with a chronic disease who seek advice from herbalists and who are taking multiple medications, as many serious adverse drug interactions between pharmaceuticals and herbs or traditional medicines have been reported [13]. While research has been done examining CAM use in patients with specific diseases and for those with one or more chronic conditions, little attention has been paid in the literature to how CAM use differs by type of chronic disease. Very few studies could be found that compare CAM utilization among individuals with different types of chronic disease; however, most were not population-based [14-17], did not adjust for other predisposing characteristics [18-20], or grouped multiple chronic conditions together [5]. One population-based United States (US) study concluded that after adjustment for demographic factors, individuals with arthritis, cancer, cardiovascular disease and lung disease were more likely to have used CAM services in the past 12 months [21]. However, due to underlying health insurance and health care systems differences between the US and other developed countries, it is unknown if these results can be extrapolated beyond the US population. This study aims to examine how CAM use differs among patients with asthma, diabetes, epilepsy and migraine compared to the general Canadian population.

## Methods

The Canadian Community Health Survey (CCHS) is a national cross-sectional survey administered to 400,055 Canadians aged ≥12 between 2001-2007, excluding those who live on Indian reserves, in institutions, in certain remote areas, or are full-time members of the Canadian Armed Forces [22]. CCHS surveys use a multistage stratified cluster design to randomly select a representative sample of Canadian household residents that represents approximately 98% of Canadians living in the provinces, 97% from the Northwest Territories, 90% from the Yukon and 71% from Nunavet [22]. Interviews are conducted in-person when possible, and self-reported information about professionally diagnosed health conditions, utilization of health services and health-related behaviours was elicited [22].

Data on chronic disease status was assessed from the following question: "Now I would like to ask you about certain chronic health conditions which you may have. We are interested in long-term conditions which are expected to last or have already lasted six months or more and that have been diagnosed by a health professional." A list of chronic conditions followed, and subjects were asked to respond yes or no. Asthma, diabetes, epilepsy, and migraine were chosen from the list of conditions in the CCHS as comparator groups for this study as they are conditions that can affect individuals throughout the life span and they are all chronic conditions that can be associated with episodic exacerbations that may require periodic acute treatment.

Data on the use of CAM services was assessed in the following way: "In the past twelve months, have you seen or talked to an alternative health care provider such as an acupuncturist, homeopath or massage therapist about your physical, emotional, or mental health?" Those who answered yes were presented with a list of options for the type of CAM provider they could have consulted. This list included: Acupuncturist, Biofeedback Teacher, Chiropractor, Feldenkrais or Alexander Teacher, Herbalist, Homeopath or Naturopath, Massage Therapist, Reflexologist, Relaxation Therapist, Religious Healer, Rolfer, and Spiritual Healer.

Data from cycles 1.1 (2001), 2.1 (2003) and 3.1 (2005) of the CCHS were merged and weighted estimates were constructed to examine the prevalence of CAM use. Statistics Canada's methodology for survey merging was followed, including the recommendation that bootstrapping not be used for merged surveys. Descriptive statistics (frequencies and crude odds ratios (ORs) with 95% confidence intervals (CIs)) were calculated and logistic regression was used to construct a multivariable model examining what factors are associated with CAM use using Stata IC version 9. Demographic factors (sex, age, education, income, marital status, residence in an urban or rural area) and disease factors (self-reported asthma, diabetes, epilepsy or migraine) were initially added to the model. Variables not achieving statistical significance at the alpha = 0.05 level were removed until all variables remaining in the model were significant.

This study was approved by the Conjoint Health Research Ethics Board at the University of Calgary.

## Results

### Baseline Demographics

Throughout the three cycles 400,055 Canadians were interviewed as part of the CCHS. As can be seen in Table 1, those with a chronic disease were more likely to be in the lowest household income bracket and to not have completed a post-secondary degree than the general population. Additionally, there were a significantly higher proportion of females in the asthma and migraine groups compared to the general population, while those with asthma or epilepsy were significantly less likely to be married or in a common-law relationship. Finally, those with diabetes were significantly more likely to live in a rural area.

**Table 1:** Demographic Characteristics of Sample

	General Population	Asthma	Diabetes	Epilepsy	Migraine
N	400,055	35,455	22,432	2,555	39,797

Age (mean ± standard deviation)	45.4 ± 20.2	39.5 ± 19.2	62.9 ± 14.7	43 ± 17.7	41.1 ± 16.8

Female Gender (%, 95% CI)	50.7 (50.5-51.0)	58.9% (58.1-59.7)	46.9 (45.9-48.0)	50.9 (47.9-53.8)	71.4 (70.7-72.1)

Marital Status (%, 95% CI)					
• Married/Common Law	58.4 (58.2-58.6)	50.3% (49.5-51.1)	67.6 (66.7-68.6)	48.0 (45.0-50.9)	59.7 (58.9-60.4)
• Widowed/Separated/Divorced	11.7 (11.6-11.8)	12.5% (12.0-12.9)	22.6 (21.8-23.4)	13.6 (11.8-15.4)	12.1 (11.6-12.5)
• Single	29.9 (29.7-30.1)	37.2% (36.4-38.0)	9.8 (9.2-10.3)	38.4 (35.6-41.2)	28.2 (27.6-28.9)

Income Quintiles (household) (%, 95% CI)					
• Lowest	14.1% (13.9-14.3)	17.6% (17.0-18.2)	26.2% (25.3-27.1)	27.1% (24.4-29.7)	15.6% (15.1-16.1)
• Low middle	17.2% (17.0-17.4)	17.3% (16.7-18.0)	21.7% (20.8-22.6)	18.4% (15.9-20.8)	16.6% (16.0-17.2)
• Middle	21.3% (21.1-21.6)	20.5% (19.8-21.2)	18.6% (17.6-19.5)	17.7% (15.0-20.3)	21.2% (20.5-21.9)
• Upper middle	23.2% (22.9-23.4)	22.0% (21.2-22.7)	13.8% (12.9-14.6)	16.9% (14.3-19.5)	23.3% (22.6-24.0)
• Highest	24.2% (23.9-24.4)	22.6% (21.9-23.4)	19.7% (18.8-20.6)	20.0% (17.4-22.6)	23.3% (22.6-24.0)

Residence (%, 95% CI)					
• Urban	81.6% (81.5-81.8)	82.2% (81.7-82.8)	80.1% (79.4-80.8)	80.9% (78.8-83.1)	82.0% (81.5-82.5)
• Rural	18.4% (18.2-18.5)	17.8% (17.2-18.3)	19.9% (19.2-20.6)	19.1% (16.9-21.2)	18.0% (17.5-18.5)

Highest Level of Education Completed (%, 95% CI)					
• Did not complete high school	26.6% (26.4-26.9)	32.4% (31.7-33.2)	38.5% (37.5-39.5)	38.4% (35.5-41.3)	24.4% (23.7-25.0)
• Completed high school	17.3% (17.3-17.5)	15.4% (14.8-16.0)	15.8% (15.0-16.5)	18.5% (16.2-20.8)	17.7% (17.1-18.3)
• Some post-secondary	8.3% (8.1-8.4)	9.3% (8.8-9.8)	5.9% (5.4-6.4)	8.1% (6.5-9.8)	8.9% (8.4-9.3)
• Completed post-secondary	47.8% (47.5-48.0)	42.8% (42.0-43.6)	39.8% (38.8-40.9	35.0% (32.1-37.9)	49.1% (48.3-49.9)

### Prevalence of CAM Use

**Figure 1:**
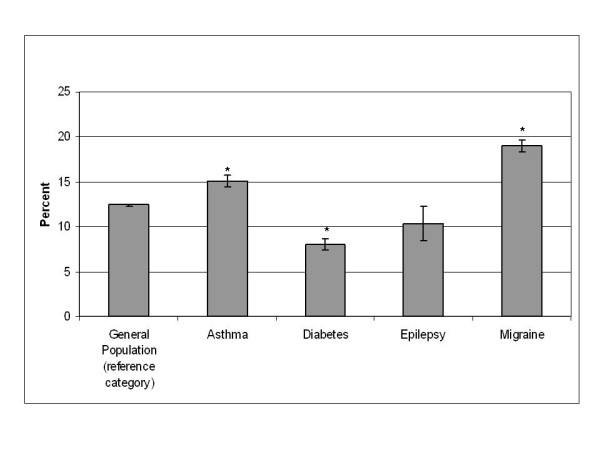
**Consulted a CAM practitioner in the past 12 months**. * significantly different from the general population

In the combined three cycles of the CCHS, 12.4% (95% CI: 12.2-12.5) of the general population reported consulting a CAM practitioner in the past 12 months (Figure 1). Prior to pooling, the data was examined to look at changes in CAM use over time for the general population and no significant differences were observed. Individuals with asthma (OR = 1.29, 95% CI: 1.23-1.36) and migraine (OR = 1.78, 95% CI: 1.71-1.86) had a significantly higher odds of using CAM services in the past year than the general population, while those with diabetes had a significantly lower odds of CAM use (OR = 0.60, 95% CI: 0.56-0.65) (Figure 1). Individuals with epilepsy (OR = 0.81, 95% CI: 0.66-1.00) had slightly lower odds of consulting a CAM practitioner than the general population; however, this did not achieve statistical significance (Figure 1). In this same time period, 77.7% (95% CI: 77.5-77.9) of the general population reported consulting a family physician, while significantly higher odds were reported for those with chronic conditions (asthma: OR = 1.75, 95% CI: 1.67-1.84, diabetes: OR = 3.20, 95% CI: 2.95-3.47, epilepsy: OR = 2.04, 95% CI: 1.70-2.46, migraine: OR = 1.72, 95% CI: 1.65-1.80).

**Figure 2:**
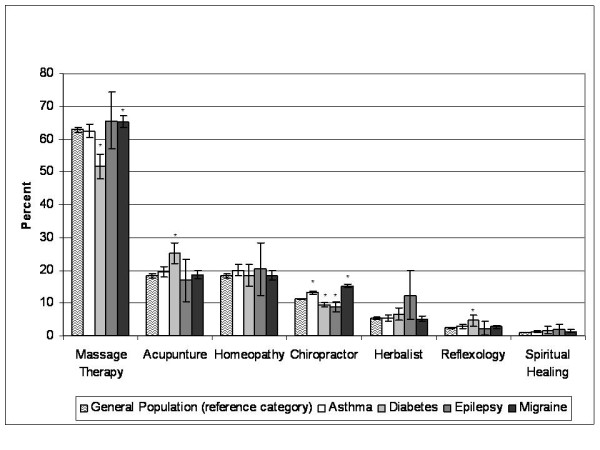
**Common types of CAM use (rates presented as a percentage of those who reported using any CAM services in the past 12 months)**. * significantly different from the general population

While various types of CAM practitioners were consulted, the most common types used by individuals who had reported visiting a CAM practitioner in the past twelve months were: massage therapy (62.9%, 95% CI: 62.2-63.6), acupuncture (18.3%, 95% CI: 17.7-18.8), homeopathy (18.2%, 95% CI: 17.7-18.8), chiropractic care (11.3%, 95% CI: 11.1-11.4), herbalists (5.2%, 95% CI: 4.9-5.6), reflexology (2.4%, 95% CI: 2.2-2.6), and spiritual healing (1.0%, 95% CI: 0.8-1.1) (Figure 2). Significant differences were found in the use of specific types of CAM practitioners based on type of chronic disease (Figure 2). Those with epilepsy (OR = 0.76, 95% CI: 0.63-0.91) and diabetes (OR = 0.81, 95% CI: 0.75-0.87) had significantly lower odds of using chiropractic services than the general population, while those with migraine (OR = 1.48, 95% CI: 1.41-1.54) and asthma (OR = 1.20, 95% CI: 1.14-1.27) had significantly higher odds. Additionally, individuals with diabetes had significantly higher odds of consulting an acupuncturist (OR = 1.54, 95% CI: 1.29-1.83) and a reflexologist (OR = 2.00, 95% CI: 1.30-3.07) in the past year than members of the general population but had lower odds of receiving a massage (OR = 0.62, 95% CI: 0.53-0.72), while those with migraine had significantly higher odds of seeing a massage therapist in the past year than the general population (OR = 1.13, 95% CI: 1.05-1.23).

### Predictors of CAM Use

Asthma, diabetes and migraine remained significant predictors of CAM use after controlling for sociodemographic factors (see Table 2). Those with asthma (OR = 1.28, 95% CI: 1.22-1.35) and migraine (OR = 1.42, 95% CI: 1.36-1.49) were significantly more likely to have used CAM services in the past year, while those with diabetes (OR = 0.80, 95% CI: 0.73-0.88) were significantly less likely to have used CAM services during this time period. A positive correlation was observed between CAM use and increasing levels of education and income. Additionally, those in the 25-44 year age group were the most likely to have used CAM services in the past year, women were more likely to have used CAM services than men, and individuals who were not currently married or in a common law relationship were more likely to have used CAM services than those with a live-in partner.

**Table 2:** Predictors of CAM Use

Variable	Odds Ratio (95% CI)
Asthma	
0 = No	Ref
1 = Yes	1.28 (1.22-1.35)

Diabetes	
0 = No	Ref
1 = Yes	0.80 (0.73-0.88)

Migraine	
0 = No	Ref
1 = Yes	1.42 (1.36-1.49)

Sex	
1 = male	Ref
2 = female	1.97 (1.90-2.04)

Age Group	
0 = 25-44	Ref
1 = 12-24	0.55 (0.52-0.59)
2 = 45-64	0.91 (0.88-0.95)
3 = ≥65	0.48 (0.46-0.52)

Highest Level of Education	
1 = didn't graduate from high school	Ref
2 = high school grad	1.59 (1.49-1.69)
3 = some post-secondary	2.03 (1.89-2.18)
4 = post-secondary grad	2.51 (2.38-2.65)

Marital	
1 = married/common law	Ref
2 = widowed/separated/divorced	1.14 (1.09-1.20)
3 = single	1.11 (1.05-1.16)

Income Quintiles (based on household income)	
1 = lowest	Ref
2 = low middle	1.17 (1.10-1.24)
3 = middle	1.35 (1.27-1.43)
4 = upper middle	1.58 (1.49-1.68)
5 = highest	1.71 (1.61-1.82)

## Discussion

This study found that approximately 12% of Canadians used some sort of CAM service in the past 12 months, and that CAM use was associated with female gender, high income and high levels of education. An inverted U-shaped relationship was noted between CAM use and age. This pattern has also been observed in other studies [8-10], indicating that CAM use may either have generational effects or be what Millar refers to in his paper as a "mid-life phenomenon" [8]. This study also shows that those with a chronic disease are not uniformly high users of CAM services. Those with epilepsy had slightly lower level of CAM use in the past year as the general population, but this did not reach statistical significance, while those with diabetes were significantly less likely to have used any CAM service in the past year. Additionally, those with different chronic conditions are consulting different CAM practitioners.

That individuals with asthma, diabetes and migraine remain statistically more (in the case of asthma and migraine) or less (in the case of diabetes) likely to utilize CAM services after adjustment for demographic factors such as age, sex and income suggests that these differences cannot be explained by predisposing characteristics alone, and in fact, represent true differences between groups. This difference may be due in part to the relatively constant nature of diabetes, once adequate blood sugar control has been achieved, as opposed to the more sporadic and acute nature of asthma and migraine attacks. A study using data from Statistics Canada's National Population Health Survey (1994-1999) found that individuals with arthritis/rheumatism, asthma, back problems, bronchitis/emphysema, Crohn's disease, and migraine were more likely to use CAM services than the general population [8]. However, once adjustments were made for chronic pain only those with asthma and back problems had significantly higher use than the general population [8]. Another study found that approximately two-thirds of survey respondents reported using CAM because they felt that conventional treatments were not effective for treating their health problem [23]. This may support the hypothesis that once adequate control of a chronic condition is achieved, individuals do not feel the need to seek out alternative treatments. Furthermore, current conventional care paradigms for diabetes tend to be holistic in nature and require patients to be actively involved in preventing further symptoms, while treatment for both migraine and asthma tends to be more reactionary when symptoms occur and focuses on pharmaceutical management.

The finding of differential use of CAM services by type of chronic disease is important as it is commonly stated in the literature that many CAM users do not disclose or discuss their CAM use with their primary care physician, and due to polypharmacy, those with chronic disease are at an increased risk of complex drug interactions [12]. A study examining herbal use in children presenting to a Canadian emergency room found that approximately 16% of children were taking medications and natural health products that could potentially interact [13]. Additionally, a survey of patients receiving care from the Canadian College of Naturopathic Medicine found that only 58.5% of respondents discussed CAM use with their primary care physician, but 90.9% discussed their prescription medication use with their naturopathic physician [12]. As this study and others show that individuals with certain chronic conditions are more likely to use CAM services, and these services are often used alongside standard medical care, this indicates that the onus has now been placed on care providers to specifically inquire about the use of CAM services. While approximately 70% of physicians do not ask their patients about the use of CAM services, physician inquiry has been shown to increase patient disclosure of CAM use by a factor of 19 (OR = 18.77, 95% CI: 5.06-69.62) [12]. Research also shows that physicians are uncomfortable discussing CAM services with their patients as generally they have little formal knowledge or personal experience with these services [24].

Ultimately, patients often seek out alternative treatments when they feel they are missing something from Canada's conventional health care system. A Canadian study examining why patients chose to use CAM services found that while the most commonly reported reasons that patients used CAM services were that these services allowed them to take a more active role in their health and they identified with the holistic approach [23]. Additionally, 40.1% of respondents reporting using CAM services because they had problems communicating with their medical doctor, and approximately two-thirds of respondents reported that conventional medicine was not effective for their particular health issue, that they were desperate, and were at a point where they were willing to try anything [23].

There are strengths and limitations to our study. The main strengths of the study include its population-based ascertainment and the very large sample size. Limitations of this study include its reliance on self-report data, and a lack of data on the type of health complaints that were addressed by CAM practitioners and rationales for seeking alternative treatments. Statistical correction for multiple testing was not undertaken, hence it is possible that a spurious association was found by chance alone. However, this is a descriptive study that is not aiming assess a specific hypothesis. Additionally, while information was collected on visits to herbalists, specific detailed information on the use of natural health products that were prescribed by a CAM practitioner or self-prescribed, which may have a greater probability of negatively interacting with standard medications, was not collected.

## Conclusion

In conclusion, Canadians appear to be using CAM services in conjunction with, not instead of, conventional care. Since scientific evidence has shown the benefits of some types of CAM, greater emphasis needs to be placed on how to better assess the use and impact of CAM services in those with chronic conditions. Physicians should also be prepared to ask and answer questions about the risks and benefits of CAM services in conjunction with standard medical care.

## Competing interests

The authors declare that they have no competing interests.

## Authors' contributions

All authors made a substantial contribution to this work. AM, NJ and SP conceived and designed the study, NJ secured access to the data, AM conducted the analysis and drafted the manuscript. All authors participated in the interpretation of data, revised the manuscript and approved the final version of the manuscript that is now being submitted for publication.

## Pre-publication history

The pre-publication history for this paper can be accessed here:

http://www.biomedcentral.com/1472-6882/10/58/prepub

## References

[B1] Boon HS Olatunde F Zick SM Trends in complementary/alternative medicine use by breast cancer survivors: comparing survey data from 1998 and 2005 BMC Womens Health 2007 7 4 10.1186/1472-6874-7-4 17397542 PMC1851951

[B2] Samdup DZ Smith RG Il Song S The use of complementary and alternative medicine in children with chronic medical conditions Am J Phys Med Rehabil 2006 85 10 842 846 10.1097/01.phm.0000233183.17059.b9 16998432

[B3] Foltz V St Pierre Y Rozenberg S Rossignol M Bourgeois P Joseph L Adam V Penrod JR Clarke AE Fautrel B Use of complementary and alternative therapies by patients with self-reported chronic back pain: a nationwide survey in Canada Joint Bone Spine 2005 72 6 571 577 10.1016/j.jbspin.2005.03.018 16256395

[B4] Park J Use of alternative health care Health Reports 2004 16 2 39 42

[B5] Harris P Rees R The prevalence of complementary and alternative medicine use among the general population: a systematic review of the literature Complement Ther Med 2000 8 2 88 96 10.1054/ctim.2000.0353 10859601

[B6] Roth M Kobayashi K The use of complementary and alternative medicine among Chinese Canadians: Results from a national survey J Immigrant Minority Health 2008 10 517 528 10.1007/s10903-008-9141-7 18386179

[B7] Quan H Lai D Johnson D Verhoef M Musto R Complementary and alternative medicine use among Chinese and white Canadians Can Fam Physician 2008 54 11 1563 1569 19005129 PMC2592333

[B8] Millar WJ Patterns of use--alternative health care practitioners Health Rep 2001 13 1 9 21 15069805

[B9] Wiles J Rosenberg MW 'Gentle caring experience'. Seeking alternative health care in Canada Health Place 2001 7 3 209 224 10.1016/S1353-8292(01)00011-9 11439256

[B10] McFarland B Bigelow D Zani B Newsom J Kaplan M Complementary and alternative medicine use in Canada and the United States Am J Public Health 2002 92 10 1616 1618 10.2105/AJPH.92.10.1616 12356610 PMC1447296

[B11] Andrews G Boon H CAM in Canada: places, practices, research Complementary Therapies in Clinical Practice 2005 11 21 27 10.1016/j.ctcp.2004.10.004 15984220

[B12] Busse JW Heaton G Wu P Wilson KR Mills EJ Disclosure of natural product use to primary care physicians: a cross-sectional survey of naturopathic clinic attendees Mayo Clin Proc 2005 80 5 616 623 10.4065/80.5.616 15887429

[B13] Goldman RD Rogovik AL Lai D Vohra S Potential interactions of drug-natural health products and natural health products-natural health products among children J Pediatr 2008 152 4 521 526 526 e521-524 10.1016/j.jpeds.2007.09.026 18346508

[B14] Cherniack EP Senzel RS Pan CX Correlates of use of alternative medicine by the elderly in an urban population J Altern Complement Med 2001 7 3 277 280 10.1089/107555301300328160 11439850

[B15] Lee GB Charn TC Chew ZH Ng TP Complementary and alternative medicine use in patients with chronic diseases in primary care is associated with perceived quality of care and cultural beliefs Fam Pract 2004 21 6 654 660 10.1093/fampra/cmh613 15531625

[B16] Cheung CK Wyman JF Halcon LL Use of complementary and alternative therapies in community-dwelling older adults J Altern Complement Med 2007 13 9 997 1006 10.1089/acm.2007.0527 18047447

[B17] Hasan SS Ahmed SI Bukhari NI Loon WC Use of complementary and alternative medicine among patients with chronic diseases at outpatient clinics Complement Ther Clin Pract 2009 15 3 152 157 10.1016/j.ctcp.2009.02.003 19595416

[B18] Ong CK Petersen S Bodeker GC Stewart-Brown S Health status of people using complementary and alternative medical practitioner services in 4 English counties Am J Public Health 2002 92 10 1653 1656 10.2105/AJPH.92.10.1653 12356616 PMC1447302

[B19] Schwarz S Messerschmidt H Volzke H Hoffmann W Lucht M Doren M Use of complementary medicinal therapies in West Pomerania: a population-based study Climacteric 2008 11 2 124 134 10.1080/13697130801930674 18365855

[B20] Eisenberg DM Kessler RC Foster C Norlock FE Calkins DR Delbanco TL Unconventional medicine in the United States. Prevalence, costs, and patterns of use N Engl J Med 1993 328 4 246 252 10.1056/NEJM199301283280406 8418405

[B21] Saydah SH Eberhardt MS Use of complementary and alternative medicine among adults with chronic diseases: United States 2002 J Altern Complement Med 2006 12 8 805 812 10.1089/acm.2006.12.805 17034287

[B22] Canadian Community Health Survey http://www.statcan.gc.ca/cgi-bin/imdb/p2SV.pl?Function=getSurvey&SDDS=3226&lang=en&db=imdb&adm=8&dis=2

[B23] Sirois FM Motivations for consulting complementary and alternative medicine practitioners: a comparison of consumers from 1997-8 and 2005 BMC Complement Altern Med 2008 8 16 10.1186/1472-6882-8-16 18442414 PMC2390516

[B24] Brown J Cooper E Frankton L Steeves-Wall M Gillis-Ring J Barter W McCabe A Fernandez C Complementary and alternative therapies: Survey of knowledge and attitudes of health professionals at a teritary pediatric/women's care facility Complementary Therapies in Clinical Practice 2007 13 194 200 10.1016/j.ctcp.2007.03.003 17631262

